# Seeding selectivity and ultrasensitive detection of tau aggregate conformers of Alzheimer disease

**DOI:** 10.1007/s00401-018-1947-3

**Published:** 2018-12-20

**Authors:** Allison Kraus, Eri Saijo, Michael A. Metrick, Kathy Newell, Christina J. Sigurdson, Gianluigi Zanusso, Bernardino Ghetti, Byron Caughey

**Affiliations:** 1 LPVD, Rocky Mountain Laboratories, NIAID, NIH, Hamilton, MT 59840 USA; 2 Department of Pathology and Laboratory Medicine, University of Kansas School of Medicine, Kansas City, KS USA; 3 Department of Pathology, UC San Diego, La Jolla, CA USA; 4 University of Verona, Verona, Italy; 5 Indiana University School of Medicine, Indianapolis, IN USA

**Keywords:** Tau aggregate, Alzheimer disease, Chronic traumatic encephalopathy, Tauopathy, RT-QuIC, Diagnosis, Biomarker, Seeds

## Abstract

Alzheimer disease (AD) and chronic traumatic encephalopathy (CTE) involve the abnormal accumulation in the brain of filaments composed of both three-repeat (3R) and four-repeat (4R) (3R/4R) tau isoforms. To probe the molecular basis for AD’s tau filament propagation and to improve detection of tau aggregates as potential biomarkers, we have exploited the seeded polymerization growth mechanism of tau filaments to develop a highly selective and ultrasensitive cell-free tau seed amplification assay optimized for AD (AD real-time quaking-induced conversion or AD RT-QuIC). The reaction is based on the ability of AD tau aggregates to seed the formation of amyloid fibrils made of certain recombinant tau fragments. AD RT-QuIC detected seeding activity in AD (*n* = 16) brains at dilutions as extreme as 10^7^–10^10^-fold, but was 10^2^–10^6^-fold less responsive when seeded with brain from most cases of other types of tauopathy with comparable loads of predominant 3R or 4R tau aggregates. For example, AD brains had average seeding activities that were orders of magnitude higher than Pick disease brains with predominant 3R tau deposits, but the opposite was true using our previously described Pick-optimized tau RT-QuIC assay. CTE brains (*n* = 2) had seed concentrations comparable to the weakest of the AD specimens, and higher than 3 of 4 specimens with 3R/4R primary age-related tauopathy. AD seeds shared properties with the tau filaments found in AD brains, as AD seeds were sarkosyl-insoluble, protease resistant, and reactive with tau antibodies. Moreover, AD RT-QuIC detected as little as 16 fg of pure synthetic tau fibrils. The distinctive seeding activity exhibited by AD and CTE tau filaments compared to other types of tauopathies in these seeded polymerization reactions provides a mechanistic basis for their consistent propagation as specific conformers in patients with 3R/4R tau diseases. Importantly, AD RT-QuIC also provides rapid ultrasensitive quantitation of 3R/4R tau-seeding activity as a biomarker.

**Electronic supplementary material:**

The online version of this article (10.1007/s00401-018-1947-3) contains supplementary material, which is available to authorized users.

## Introduction

Alzheimer disease (AD) afflicts 5.7 million people in the US alone and their care is estimated to cost $232 billion annually (http://www.Alz.org). A key neuropathological feature of AD and other diseases involving tau pathology is the accumulation of the protein tau in the form of self-seeding filaments or sub-filamentous deposits [6]. The structures of the tau filaments of AD and Pick disease (PiD) have recently been shown to be distinct linear assemblies of tau molecules with parallel in-register intermolecular β-sheet amyloid architectures [12–14]. These are the first available structures of disease-associated tau aggregates, but they are unlikely to represent all pathological tau aggregates, because multiple permutations (referred to as strains) of tau aggregates have been isolated biologically and shown to propagate consistently in cell culture or in vivo [7, 23, 31]. Thus, distinct conformations of tau assemblies that, like prion strains, are capable of conformationally faithful replication appear to contribute to the phenotypic diversity of tau pathologies.

AD can be difficult to firmly diagnose and differentiate from other neurodegenerative diseases prior to post-mortem neuropathological examination. An ability to quantitate AD-associated tau aggregates as biomarkers with sufficient sensitivity and specificity may facilitate AD diagnosis and the monitoring of specific therapeutic targets. Indeed, the new NIA-AA Research Framework advocates the development of biological, biomarker-based, rather than primarily syndromal, definitions of AD, and their diagnostic and prognostic implications [22].

AD brain samples can seed the cell-free assembly of amyloid fibrils from recombinant tau, or fragments thereof [27, 29], but the extent to which such cell-free reactions might be useful for the detection and discrimination of different disease-associated tau aggregates has not been reported. AD brain extracts can also seed tau aggregation in cell cultures expressing fluorescently tagged tau constructs, serving as a highly sensitive assay for tau seeds [15, 19, 44]. However, the practicality of this assay for routine diagnostic purposes is limited by the need for tissue cultures and flow cytometry.

We recently developed an ultrasensitive cell-free assay for the tau aggregates of Pick disease [37]. PiD is characterized by the predominant accumulation of the human tau isoforms with three microtubule binding repeats (3R). Humans normally express six tau isoforms which differ in the inclusion of amino-proximal inserts and either 3 or 4 microtubule binding repeats (3R and 4R tau isoforms, respectively). In AD and chronic traumatic encephalopathy (CTE), comparable proportions of 3R and 4R tau isoforms are aggregated in the brain [21, 46]. Other diseases such as corticobasal degeneration (CBD), argyrophilic grain disease (AGD), and progressive supranuclear palsy (PSP) accumulate predominantly 4R tau aggregates.

Our PiD assay prototype, hereafter designated as 3R tau real-time quaking-induced conversion (3R tau RT-QuIC), takes advantage of the ability of PiD tau aggregates to seed the fibrilization of a vast stoichiometric excess of recombinant tau-derived monomers (i.e., substrates). The assay is performed in multi-well plates, and the fibrillar products are detected using an amyloid fibril-sensitive fluorescent dye, thioflavin T (ThT) [37]. 3R tau RT-QuIC, which uses a 3R tau fragment (K19CFh) as the substrate, has strong selectivity for the 3R tau aggregates of PiD over the predominantly 4R or mixed 3R/4R tau aggregates of other diseases.

Analogous RT-QuIC assays for prion diseases (reviewed in [1, 5, 35, 40, 48]) can amplify prion seeding activity by a billion-to-trillion-fold, allowing detection of seeding activity in individuals’ cerebrospinal fluid (CSF) [1, 8, 26, 33], nasal brushings [32, 47], urine [28], and skin [36]. Such testing can provide intra vitam diagnoses that can be virtually 100% sensitive and specific [2, 34]. We and others have recently described α-synuclein RT-QuIC and closely related assays for the CSF-based early diagnosis of Parkinson disease (PD) and Lewy body dementia (LBD) [11, 18, 41]. With respect to AD specifically, a significant development was the report of a seed amplification assay (called Aβ-PMCA) for Aβ oligomers, which are another key feature of AD pathogenesis [38]. When applied to CSF specimens, Aβ-PMCA gave an overall diagnostic sensitivity of 90% and specificity of 92%. Given that both Aβ and tau deposition are core features of AD pathology, it would likely be helpful in research and diagnostics to also have an AD-selective tau seed amplification assay to complement Aβ-PMCA in measuring the key causative biomarkers of AD.

Here, we describe a highly sensitive and selective tau RT-QuIC assay (AD RT-QuIC) that preferentially detects AD- and CTE-associated 3R/4R tau seeding activity over the tau seeding activity associated with diseases with tau aggregates that are predominantly composed of either 3R or 4R isoforms.

## Materials and methods

### Brain tissue samples

De-identified post-mortem brain samples were obtained from sources indicated in Online Resource Table 1 and in “Acknowledgements”.

### Preparation of human brain tissue homogenates

10% w/v brain homogenates were prepared by taking several representative sections from frozen brain sections and homogenizing in ice-cold PBS using 1 mm zirconia/silica beads (BioSpec Products, cat. no. 11079110z) and a mini Beadbeater (BioSpec) or BeadMill 24 (Fisher Scientific).

### Neuropathology

Neuropathology specimens were diagnosed by board-certified neuropathologists as indicated in Online Resource Table 1. Brain samples from B.G. were handled and evaluated neuropathologically as follows: half of the brain from affected individuals and controls was fixed in formalin and the other half was frozen. Tissue samples for neuropathological studies were obtained from representative brain regions. The following methods were used: Weigert’s hematoxylin–eosin, Woelcke–Heidenhain, Bodian, Gallyas, and thioflavin S. For immunohistochemistry, antibodies against tau, Aβ, glial fibrillary acidic protein (GFAP), prion protein, ubiquitin, and TAR DNA-binding protein-43 (TDP-43) were used. For neuropathologic diagnosis, criteria established for AD, FTLD, PD, and other neurodegenerative diseases were used [3, 4, 20, 25]. CTE samples were those characterized as described previously [39].

### Genetics

For genetic analysis, genomic DNA was extracted from fresh brain and sequenced, using standard protocols [30].

### Protein expression and purification

K19CFh was prepared as described previously [37]. Another tau construct used in the study was designed to include the core part of the AD fibril [14], with a point mutation at residue 322 cysteine to serine called τ306 (residues 306–378 using the numbering for full-length human tau isoform htau40). A stop codon was added at C terminal residue 379. The mutated cloning cassette was synthesized and cloned into a bacterial expression vector pET-28a right after the 5′ N-terminal poly-histidine tag and thrombin site by GenScript using CloneEZ seamless cloning technology.

Both constructs were expressed in BL21(DE3) *Escherichia coli* following the protocol described in [37]. Briefly, expression was induced using the Overnight Express autoinduction method [42]. Cells were pelleted at 3750 rpm for 35 min at 4 °C and resuspended and lysed in buffer A (10 mM Tris, pH 8.0, 500 mM NaCl, 5 mM imidazole), sonicated for 3 min (3 × 45 s sonication, 15 s pause). The lysate was centrifuged at 10,000×*g*, for 1 h at 4 °C and filtered through a 0.45 µm syringe filter and purified through a 5 mL His-Trap FF (GE Healthcare 17-5255-01) column. Prior to elution of τ306, the column was washed with seven column volumes of 30 mM imidazole in 10 mM Tris, pH 8.0, 500 mM NaCl, and then five column volumes of 46 mM imidazole to elute contaminants (see Online Resource Fig. 1). τ306 was eluted during a linear gradient of 46–200 mM imidazole over eight column volumes. 2 mL fractions were collected and 2 µL of 2 M DTT was added to each fraction for a final concentration of 2 mM prior to SDS-PAGE analysis. Based on SDS-PAGE analysis of purity, fractions were pooled and precipitated in four volumes of acetone overnight at 4 °C. Precipitant was centrifuged at 10,000×*g*, 20 min, 4 °C. The acetone was discarded and pellets washed with 5 mL acetone containing 2 mM DTT per 2 mL fraction. Pellets were dissolved in 8 M GdnHCl, 2 mL per fraction, and desalted over PD-10 desalting column (GE Healthcare, 17-0851-01) in 1X PBS, pH 7.0 according to the gravity protocol provided by the manufacturer. Protein concentration was determined by OD readings at 280 nm for each 0.5 mL fraction from desalting, and fractions were pooled to maximize protein yield while avoiding the addition of guanidine-containing fractions to the final pool. Protein was adjusted to 0.75 mg/mL in 1X PBS, pH 7.0 for storage at − 80 °C until use. At least five independent preparations of τ306 and K19CFh were analyzed for reproducibility in the AD RT-QuIC reaction conditions.

### AD RT-QuIC

Reaction conditions included 10 mM HEPES, pH 7.4, τ306 and K19CFh at a 1:3 molar ratio for a final total substrate concentration of 12 µM, 400 mM NaCl, 40 µM heparin (Celsus Laboratories Inc., MW 4300 Da), and 10 µM ThT. One silica bead (800 μm, Ops diagnostics) was added to each well. Reactions were adjusted for sample volume (1–2 µL) to a final volume of 50 µL per well in a 384 well plate or 100 µL in a 96 well plate. Brain homogenate samples were serially diluted in sample diluent buffer (10 mM HEPES pH 7.4, 1× N2, 0.526% brain homogenate from tau-free mouse brain homogenate). Reactions were incubated at 37 °C and shaken in cycles of 1 min orbital at 500 rpm and 1 min rest on a BMG Fluostar platereader. ThT fluorescence was measured every 45 min (450 ± 10 nm excitation, 480 ± 10 nm emission, bottom read).

### Transmission electron microscopy

Fibril solutions were collected from RT-QuIC reactions after 16 h of incubation. To collect solutions, a pipet tip was used to vigorously scrape the well surfaces and pipet the solution. 2–8 wells were pooled for each reaction condition and the solutions briefly sonicated. Ultrathin carbon on holey carbon support film grids (400 mesh, Ted Pella) were briefly glow-discharged before being immersed into droplets of the fibril solutions for 30–60 min at room temperature. Grids were sequentially washed three times in MilliQ water before being negatively stained with Nano-W (methylamine tungstate) stain (Nanoprobes, #2018) and wicked dry. Grids were imaged at 80 kV with a Hitachi H-7800 transmission electron microscope and an XR-81 camera (Advanced Microscopy Techniques, Woburn, MA).

### ATR–FTIR

RT-QuIC reaction products were recovered from 384 well plates by scraping the bottom of the well with a pipette tip and transferring the contents of 16 replicate reactions seeded with 1 × 10^−3^ dilutions of six individual sporadic AD (sAD 1–6) and three individual familial AD (fAD 1–3) brain homogenates. Reactions contained identical conditions to those described in the AD RT-QuIC section, and were stopped when ThT fluorescence reached a plateau at 15 h, prior to spontaneous fibrillization in KO-seeded reactions. Pooled samples were centrifuged at 20,800×*g* for 1 h, 4 °C, supernatant discarded and pellets washed in 200 µL D_2_O with another centrifugation at 20,800×*g* for 10 min, 4 °C. The final pellet was resuspended in ~ 5 µL D_2_O for FTIR analysis. 1.5 µL of pellet-D_2_O slurry was applied to a Perkin Elmer Spectrum 100 FTIR with diamond crystal ATR attachment. The samples were partially dried such that the 2400 cm^−1^ D_2_O band reached ~ 80% transmittance to avoid over-drying. For each sample, 100 scans were averaged from 4000–800 cm^−1^, 4 cm^−1^ step, strong apodization, with continuous purge of sample and electronic chambers with dry air. Spectra with excess contribution from water vapor were discarded and repeated. Spectra were normalized to amide I intensity and second derivative spectra were taken with nine points for slope analysis.

### Proteinase K digestion

Brain homogenates were incubated with 50 μg/mL proteinase K for 30 min at 37 °C. PK digestion was halted by incubating the homogenates on ice with 1 mM Pefabloc for 5 min. PK digestion was confirmed by gel analysis and seeding activity of protease-resistant tau assessed in the AD RT-QuIC.

### Preparation of mouse tau-free brain homogenates

Tau-free mice [B6.129S4(Cg)-Mapt^tm1(EGFP)Klt^/J] were ordered from Jackson Laboratories. Homogenates were prepared from flash-frozen brain tissue as previously described, with the exception that protease inhibitors can be included, but are not necessary for homogenate preparation [37]. All mice were maintained under pathogen-free conditions at an American Association for the Accreditation of Laboratory Animal Care accredited animal facility at the NIAID and housed in accordance with the procedures outlined in the Guide for the Care and Use of Laboratory Animals under an animal study proposal approved by the NIAID Animal Care and Use Committee (ASP # 2016-058).

### Collection of RT-QuIC products and SDS-PAGE analyses

RT-QuIC products were collected from 8 to 16 individual wells of an AD RT-QuIC plate by scraping the wells with a pipet tip and pipetting up and down before pooling the reactions in a microfuge tube. Aliquots of the total reaction were saved before centrifuging at 20,800×*g* for 20 min to 1 h. The pellet fractions were washed with 1 mL H_2_O 2–3 times prior to analysis. 5X the total concentration of the pellet fractions was loaded on the gel compared to the total reaction to visualize K19CFh and τ306. Samples were brought up in sample buffer (125 mM Tris–HCl pH 6.8, 5% glycerol, 6 mM EDTA, 10% SDS, 0.04% Bromophenol Blue, 6 M Urea, 8% β-mercaptoethanol) and boiled for 10 min. Equal volumes of each sample were run on 10% or 12% Bis–Tris NuPAGE gels (Invitrogen) and stained with GelCode Blue protein stain (ThermoFisher Scientific, 24590) per manufacturer’s instructions.

### Sarkosyl extraction

Sarkosyl-insoluble extracts were generated from brain homogenates as previously described [37]. The extracts were diluted in sample diluent buffer as needed to be compared as brain equivalents to the starting brain homogenate material, and both the sarkosyl-insoluble material and brain homogenates compared on the same 384 well plate.

### Generation of Aβ42 oligomers

Human Beta amyloid (1–42) (California Peptide Research) was dissolved in 1,1,1,3,3,3-hexafluoro-2-propanol (HFIP) and incubated at room temperature for 1 h before HFIP was evaporated overnight at RT or under N_2_ gas. To resolubilize the peptide film, DMSO was added to reach a concentration of 5 mM. The Aβ42-DMSO stock was diluted into DMEM/F12 medium without phenol red to a concentration of 100 μM and incubated at room temperature for 16 h. Aβ42-oligomers were spun at 14,000×*g* for 15 min, the supernatant aliquoted and snap frozen in liquid N_2_ before storage at − 80 °C until use. Aβ42 oligomers were verified by transmission electron microscopy, size exclusion chromatography, and western blot analysis using the 6E10 antibody.

### Preparation of synthetic tau fibrils

Synthetic tau fibrils were prepared by adding 3 µM τ306, 9 µM K19CFh, 40 µM heparin, and 10^−3^ dilutions of AD brain homogenate to a 500 µL microfuge tube and shaking the tubes continuously at 1000 rpm, 37 °C for 20 h. Coomassie gel analysis of volume-matched pellet and supernatant indicated that > 30% of the total tau was aggregated.

### Immunoprecipitation

Dynabeads Protein G Immunoprecipitation kit (10003D, ThermoFisher Scientific) was used to perform immunoprecipitation as directed by the manufacturer’s protocol with minor modifications. Briefly, 0.75 mg of beads were bound to 2 µg of anti-tau antibody HT7 (MN1000, ThermoFisher Scientific) or IgG control (14-4714-81, ThermoFisher Scientific) in 0.01% bovine serum albumin (BSA), 1× phosphate buffered saline (PBS) pH 7.4 (PBS-B) for 15 min with constant rotation at room temperature. Non-specific binding to beads was blocked by BSA. After washing with 200 µL of PBS-B, 100 µL of 0.01 or 0.001% (w/v) in PBS-B was incubated with bead–antibody complexes (2 μg antibody per 100 μL reaction) for 26 min at constant rotation at room temperature. Bead–antibody–antigen complexes were isolated with a magnet, and the supernatant (immunodepleted sample) was saved to test in AD RT-QuIC. Bead–antibody–antigen complexes were resuspended in 20 µL of elution buffer (non-denaturing) and incubated for 2 min at room temperature. Bead–antibody complexes were isolated from the eluant on the magnet, and the eluant (immunoprecipitated tau) was tested by AD RT-QuIC.

### Lag time and Spearman Kärber SD_50_ analyses

Assay cutoff was determined to be 30 h as a reproducible cut-off time before spontaneous amyloid formation in the presence of mouse tau KO brain homogenate. Positive wells were determined as those whose ThT fluorescence values exceeded 100× the standard deviation of the baseline before the assay cutoff. These values were used to determine lag time and for Spearman–Kärber analyses [10].

## Results

### Development of an AD RT-QuIC assay

**Fig. 1:**
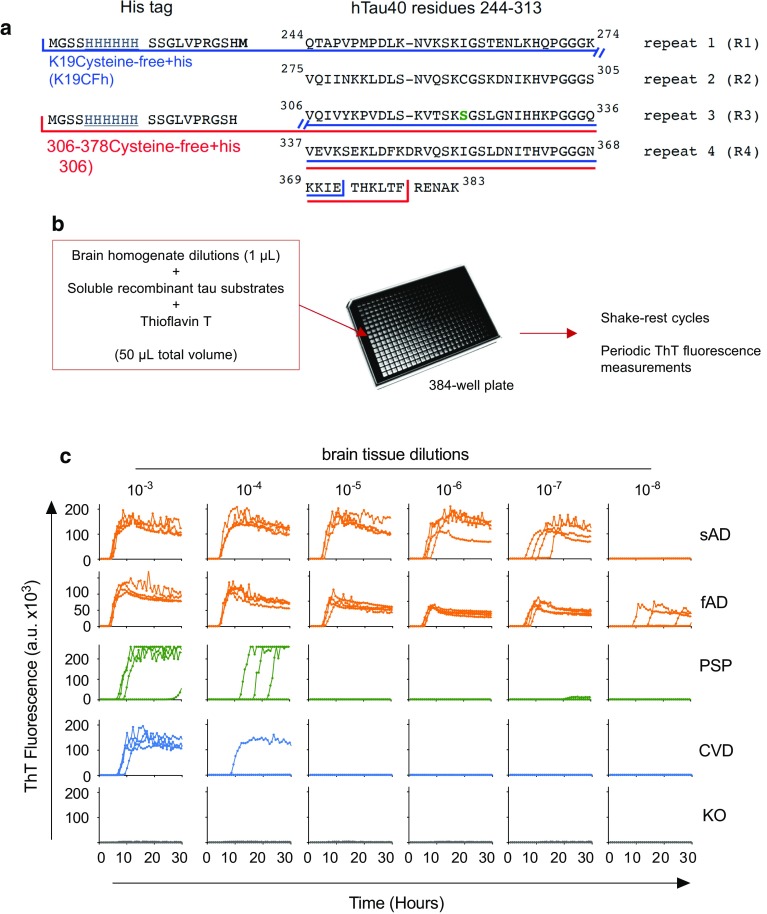
AD RT-QuIC dilution analyses of AD (3R/4R tauopathy), PSP (4R tauopathy), CVD (histologically negative for tau pathology), and KO (tau-free) brain homogenates. **a** Schematic of τ306 and K19CFh with His-tags and the S322C mutation denoted, with numbering based on the full-length htau40 sequence. **b** Reactions were seeded with 1 µL of brain homogenate at the indicated dilution in a 384-well plate, subjected to cycles of shaking and rest, and periodically measured for relative ThT fluorescence over 30 h. **c** Each curve represents an individual well, run in quadruplicate for each dilution

The amyloid cores of AD tau filaments were recently shown to be comprised of tau residues 306–378 using the numbering of the longest human tau isoform, htau40 [14]. Based on these cores, we designed a recombinant substrate (called τ306) comprised of residues 306–378 (Fig. 1a; purification, Online Resource Fig. 1). Using τ306 and previously described 3R tau RT-QuIC conditions [37], we saw more rapid seeding with 10^−3^ or 10^−4^ dilutions of AD brain homogenate relative to 10^−3^ dilutions of brain tissue that showed no tau pathology and was being used as control tissue harvested from individuals diagnosed as having Diffuse Lewy body Dementia (DLBD) (Online Resource Fig. 2a, c). The addition of a threefold stoichiometric excess of a second tau-based substrate, K19CFh (Online Resource Fig. 2b, d), slowed the AD-seeded reactions, but allowed a more dilute AD homogenate (10^−5^) to be discriminated from homogenates prepared from brains that were found to be free of tau pathology in immunohistochemical analyses (Online Resource Fig. 2b, d). Focusing on a 1:3 stoichiometric ratio of τ306:K19CFh substrates, we varied multiple reaction conditions, as shown in part in Online Resource Fig. 3. Our optimal conditions for distinguishing brains with AD pathology from those with cerebrovascular disease (CVD) or other pathologies, or from brains of tau knock-out (KO) mice, are those described as “AD RT-QuIC” in “Methods”. These conditions allowed detection in 10^−7^–10^−10^ dilutions of our initial single examples of sporadic (sAD) and familial (fAD) AD brain specimens in 30 h reactions (Fig. 1). In contrast, no responses were seen in reactions seeded with KO brain within this timeframe. Although some positive reactions were seen with more concentrated CVD and PSP brain specimens, these seeding activities became undetectable in dilutions beyond 10^−4^.

**Fig. 2:**
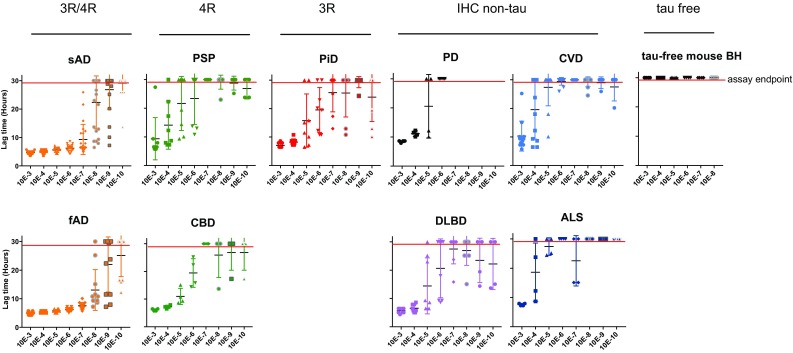
Lag time analysis of AD RT-QuIC reactions seeded with dilutions of AD and non-AD brain homogenates. Lag time was determined as the reaction time required to exceed a ThT fluorescence threshold of the average + 100 standard deviations of the baseline fluorescence. Symbols indicate lag times from individual wells. Cross hatches and bars indicate the mean ± SD of the values at each dilution. The assay endpoint was 30 h, and thus, any data points beyond the red line had positive ThT fluorescence values at or greater than 30 h. A value of 30 was assigned to data points beyond the assay endpoint to calculate mean ± SD

In further experiments, we found that the lag phase prior to the threshold ThT fluorescence was shorter in AD RT-QuIC reactions seeded with sAD or fAD brain specimens compared to comparable dilutions of non-AD brain tissue (Fig. 2). This indicated higher specific seeding activities (per unit of tissue) in the AD brains.

### Quantitation of seeding activity associated with AD and non-AD brains

**Fig. 3:**
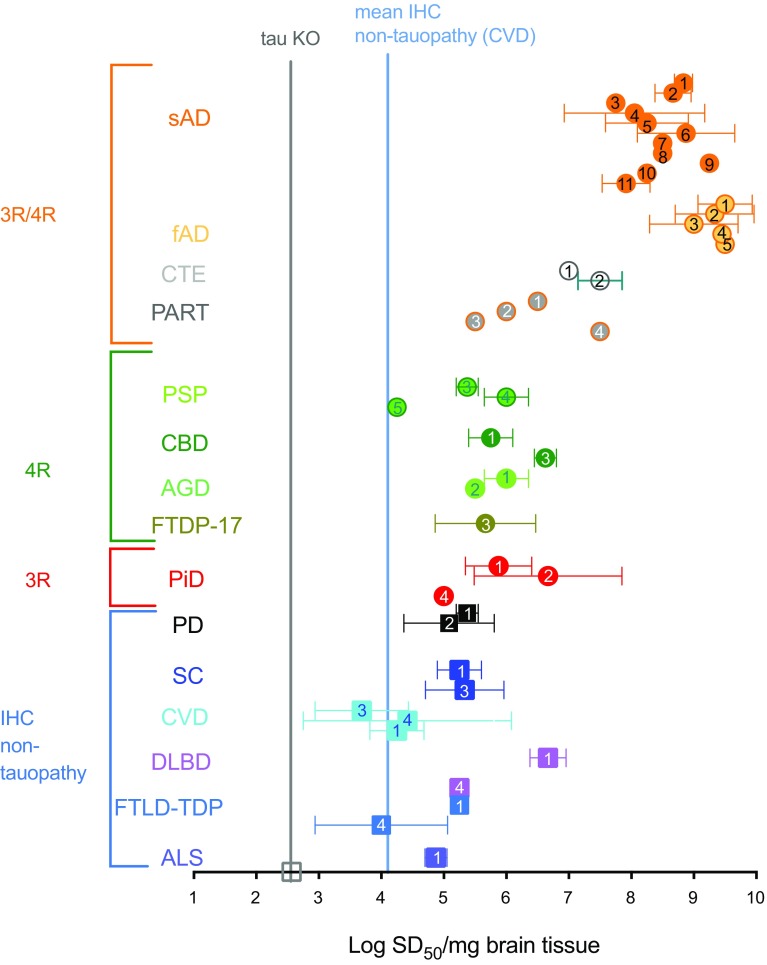
AD RT-QuIC end-point dilution analysis of brain homogenates from cases of AD, CTE, and other neurological disorders. The seeding dose (SD_50_) was determined by Spearman–Kärber analyses and is shown as log SD_50_/mg brain tissue. The vertical grey and blue lines mark the average values from brains of tau knock-out (KO) mice and human CVD cases lacking immunohistochemical evidence of tau pathology, respectively. *sAD* sporadic AD, *fAD* familial AD, *CTE* chronic traumatic encephalopathy, *PiD* Pick disease, *PSP* progressive supranuclear palsy, *CBD* corticobasal degeneration, *AGD* argyrophilic grain disease, *FTDP-17* frontotemporal dementia and Parkinsonism-17, *SC* senile changes (non-tau associated), *CVD* cerebrovascular disease, *DLBD* diffuse Lewy body disease, *FTLD-TDP* frontotemporal lobar degeneration with TDP-43, *ALS* amyotrophic lateral sclerosis, *PD* Parkinson disease, *IHC* immunohistochemistry. Data are represented as mean ± SD. sAD, fAD, p < 0.0001; CTE, p < 0.0001; PART, p=0.0002; CBD, p = 0.005; AGD, p=0.04, PiD, p=0.01; DLBD, p=0.015 by one-way ANOVA [F(15, 31) = 29.15] compared to CVD

We then assayed numerous additional AD and non-AD brain specimens, including two CTE brain samples from boxers (Online Resource Table 1). We quantified relative seeding activities using end-point dilutions and Spearman–Kärber estimation of the tissue equivalent giving positive AD RT-QuIC reactions in 50% of replicates, i.e., the 50% seeding “dose” or SD_50_ [37, 45]. The SD_50_ concentrations in sporadic AD (sAD) cases averaged (± SD) 8.4 ± 0.7 log SD_50_/mg tissue (*n* = 11) (Fig. 3). The concentrations in familial AD (fAD) cases (*n* = 5) were higher at 9.4 ± 0.5 log SD_50_/mg tissue (*p* = 0.01, unpaired, two tailed *t* test). CTE brain samples (*n* = 2, temporal cortex) overlapped the low end of the range observed for sAD samples, with averages of 7.0 and 7.5 log SD_50_/mg tissue. Notably, these AD and CTE seeding activities were 0.5–3 orders of magnitude higher than those for all but one of the designated non-AD brain specimens (Fig. 3). Particularly striking was the comparison of the AD cases to the PiD cases, which in the 3R tau RT-QuIC assay, had 4–5 logs higher seeding activity than AD cases [37]. Previous analyses indicated that one of these specific AD specimens and three of the PiD specimens contained similar (within threefold) loads of sarkosyl-insoluble tau aggregates [37]. Thus, the marked inversion of relative seeding activities of the AD and PiD brain specimens using the AD and 3R tau RT-QuIC assays indicated major qualitative, rather than merely quantitative differences between AD and PiD tau seeds. Overall, these results indicated strong, but perhaps not absolute, selectivity of the AD RT-QuIC conditions for seeding activity associated with AD.

We assayed brain tissue from four cases of primary age-related tauopathy (PART), a condition associated with 3R/4R tau deposits. One PART case (i.e., PART 4) had seeding activity that overlapped the low end of the AD range. Other than PART 4, seeding activities measured in the PART samples were largely comparable to seeding activities measured in non-AD brain specimens, suggesting either a quantitative discrimination between 3R/4R tau pathologies of AD and PART, or an absence of tau pathology in brain regions tested.

### Immune capture of seeding activity with tau antibodies

**Fig. 4:**
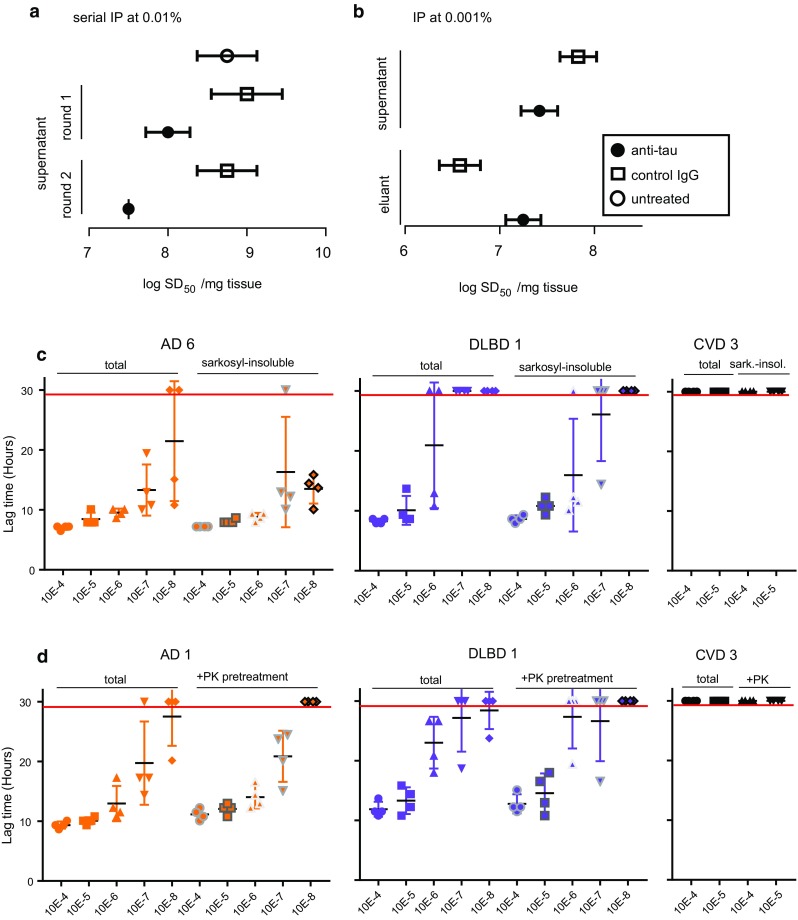
Tau antibody reactivity, detergent insolubility, and protease resistance of AD seeding activity. **a** Serial immunodepletion. HT7 anti-tau and control IgG antibody-conjugated beads were incubated with sAD brain homogenate and two rounds of unbound (supernatant) fractions were assayed by end-point dilution in the AD RT-QuIC. **b** Immune capture. Bound (then eluted from beads) and unbound (supernatant) fractions of sAD brain homogenate were assayed by end-point dilution in the AD RT-QuIC. Mean log SD_50_ values (± SE) were calculated using Spearman–Kärber analysis. **c** Dilution analysis of total and sarkosyl-insoluble fractions derived from identical brain equivalents of CVD, DLBD, and AD brain homogenates. Lag times from individual reactions seeded with the designated dilutions are shown. Horizontal bars indicate the mean ± SD of quadruplicate lag times. **d** AD, DLBD, and CVD brain homogenates were digested with proteinase K (+PK) and used for dilution analysis. Lag times are shown for untreated (total) and proteinase K-treated brain homogenates. For **c**, **d**, any data points beyond the assay endpoint (indicated by a red line) had positive ThT fluorescence values at or greater than 30 h. These data points were assigned a value of 30 to calculate mean ± SD

To confirm that the seeding activity detected in AD brain homogenates contained tau, we performed seed capture experiments using beads bound with either tau antibody HT7 or an isotype-matched control antibody (Fig. 4a, b). Supernatants of 10^−4^ dilutions of AD brain homogenate incubated with HT7 showed an 82% reduction in seeding activity following one round of immunodepletion, and another 12% reduction in seeding activity following a second round of immunodepletion, while incubation with control IgG beads showed no reduction in seeding activity (Fig. 4a). In other independent experiments at 10^−5^ (Fig. 4b) and 10^−6^ (not shown), depletions of 62% and 69%, respectively, were seen with the HT7 antibody relative to the control antibody in single-round immunoprecipitations, while corresponding eluates from the HT7 immunoprecipitates had 3.2–4.5-fold higher seeding activities than the control IgG immunoprecipitates. Overall, these results indicated that most of the seeding activity (up to 94% of original) could be captured with anti-tau coated beads.

### Detergent insolubility and protease resistance of brain-derived AD RT-QuIC-seeding activity

For further confirmation that the AD RT-QuIC detects tau aggregates, we determined that, like the tau filaments of AD and other diseases with tau pathology [16], AD-associated seeding activity was sarkosyl-insoluble and proteinase K-resistant (Fig. 4c, d, Online Resource Fig. 4a–d). Insoluble and protease-resistant seeding activity was detected in AD brain tissues as well as in a DLBD brain sample with relatively high seeding activity (DLBD 1, ~ 10^7^ SD_50_/mg tissue), but not in CVD brain tissue. Given that AD, PSP, CBD, and PiD brain tissue can have similar (within ~ fivefold) loads of aggregated, sarkosyl-insoluble tau [37], it is likely that the logarithmically higher AD RT-QuIC-seeding activities in AD brains were largely due to the characteristics, rather than the quantity, of tau aggregates.

### AD tau detection was not impacted by Aβ42 oligomers

As Aβ42 oligomers accumulate early in AD, their co-occurrence with tau amyloid in AD brain homogenates might have cross-seeding or inhibitory effects in our AD RT-QuIC assay. To test this, Aβ42 oligomers were prepared from synthetic peptides and mixed at different ratios with AD or mouse tau KO brain homogenates (Online Resource Fig. 4e). Inclusion of Aβ42 oligomers with AD brain homogenates did not influence the kinetics or sensitivity of detecting seeding activity from AD brain homogenates. In addition, no increased tau fibrillization was detected when Aβ42 oligomers were added to KO brain homogenates, suggesting that there were no significant tau cross-seeding effects from Aβ42 oligomers under the AD RT-QuIC conditions (Online Resource Fig. 4e).

### Characterization of products of AD RT-QuIC reactions

**Fig. 5:**
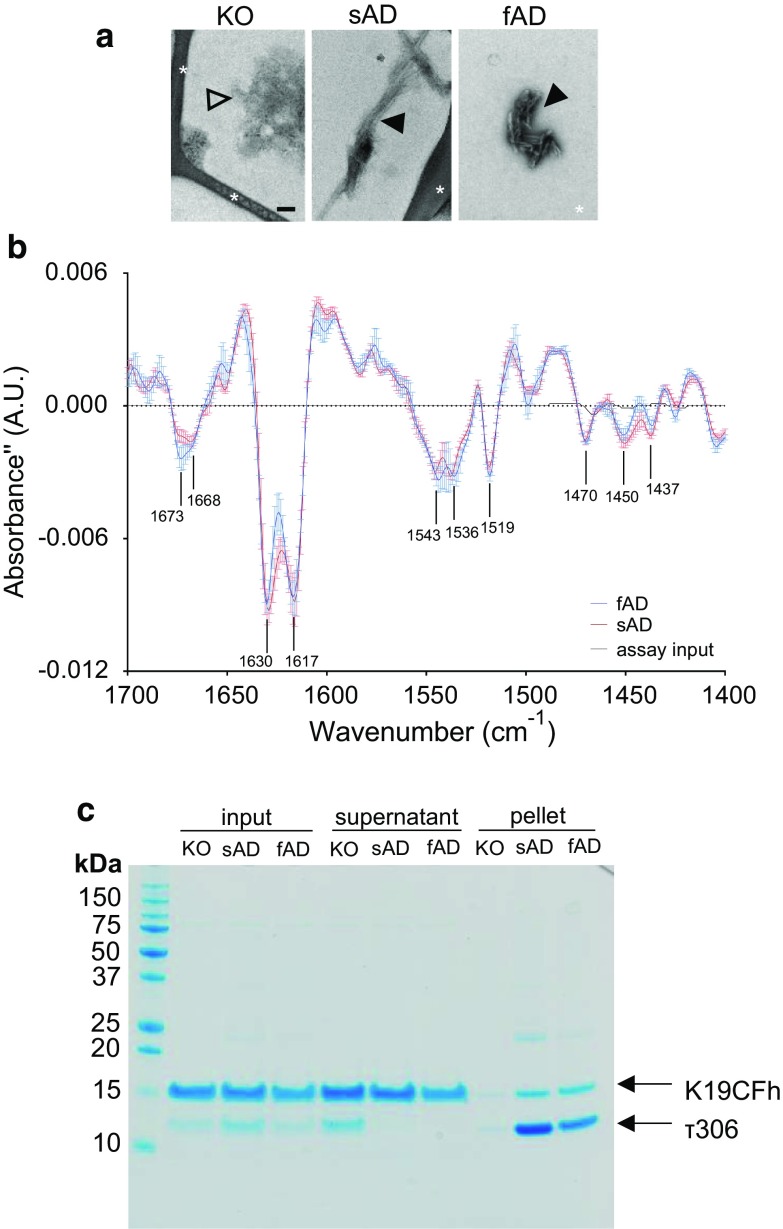
AD RT-QuIC products are fibrillar, high in β-sheet and insoluble. **a** Negative stain electron micrographs of products of AD RT-QuIC reactions seeded with KO, sAD or fAD brain homogenates. Open arrow head: amorphous aggregates in KO-seeded reactions; Closed arrowheads: fibrils in AD-seeded reactions. Asterisks: EM grid structure. **b** Average ± SD of second derivative FTIR spectra of proteinase K-treated RT-QuIC substrate input and recovered products are shown. RT-QuIC products used for analysis were initially seeded with fAD or sAD as indicated. Prominent bands at 1630 and 1617 cm^−1^ are consistent with β-sheet secondary structure. **c** RT-QuIC products were collected after 20 h of incubation, pelleted, and compared to the starting reaction (input) and supernatant fractions using gel analysis. Pellets were concentrated ~ fivefold compared to the total reaction. K19CFh and τ306 are denoted with arrows

We then analyzed the products of the AD RT-QuIC reactions. The following characteristics were consistent with them being amyloid fibrils: (1) the enhancement of ThT fluorescence (Fig. 1); (2) fibrillar ultrastructures by transmission electron microscopy (Fig. 5a); (3) prominent infrared absorbance bands at 1630 and 1617 cm^−1^ indicating β-sheet secondary structure composition [9, 17, 43] (Fig. 5b); and (4) sarkosyl-insolubility and proteinase K-resistance (Fig. 4c, d, Online Resource 4). SDS-PAGE gel analyses of the insoluble products (see pelleted fraction) from reactions seeded with sAD or fAD brain homogenates indicated the presence of both τ306 and K19CFh substrates (Fig. 5c). However, despite the threefold excess of K19CFh over τ306 in the reaction mixture, τ306 was preferentially incorporated into the insoluble fraction, indicating a strong preference for incorporation of τ306 in forming the ThT-positive aggregates seeded by AD brain homogenates.

### Analytical sensitivity

It is difficult to accurately quantitate the total concentrations (mass per unit tissue) of tau species with seeding activity in tissue specimens because of their potential heterogeneity in sequence and size that likely ranges from small soluble oligomers to large insoluble filaments. However, average overall tau levels in AD brain have been reported to be ~ 7 ng/µg protein [24], which converts to ~ 10 µM in solid brain tissue. Our ability to detect seeding activity in 10^−7^–10^−10^ dilutions of AD brains suggests that our minimal detectable concentration of AD-associated tau seeds (which must be a subset of total tau) in a test sample should be ≤ 1–1000 fM (based on monomer concentration) or 0.05–50 fg per 1 µL test sample. As another imperfect approach to estimating the analytical sensitivity, we generated synthetic amyloid products of AD-seeded AD RT-QuIC assays, with known total concentration of the tau molecules, and used them as surrogate seeds in end-point dilution AD RT-QuIC assays. The minimum detectable amount of these synthetic tau seeds was 1.4 pM or ~ 16 fg in a 1 µL test sample (Online Resource Fig. 5). Overall, these estimates match or exceed estimated sensitivities of cell-based assays for tau-seeding activities [19].

### Detection of AD seeds at different Braak stages

**Fig. 6:**
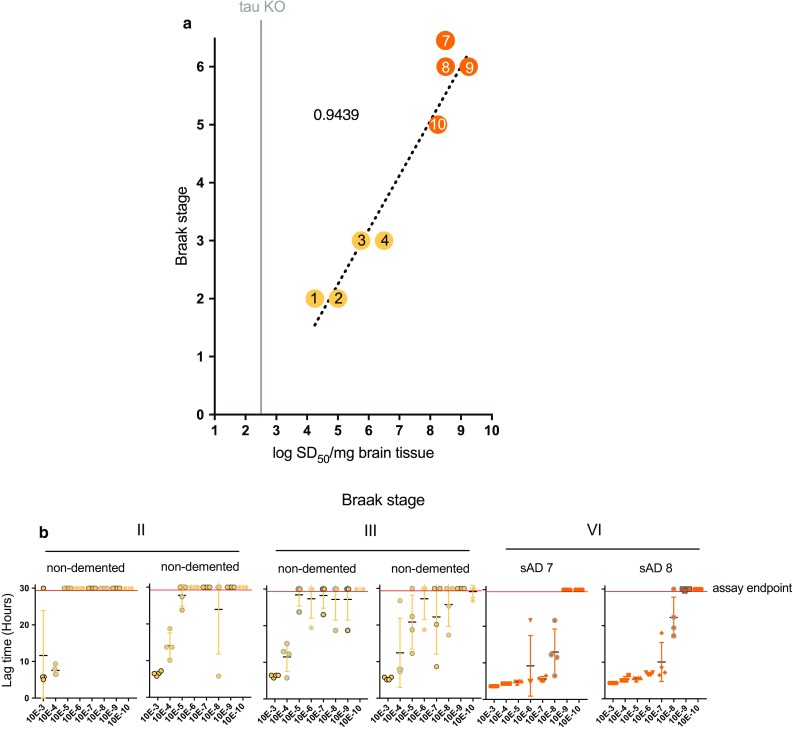
AD RT-QuIC dilution analyses of cortex tissue homogenates from brain samples with different Braak stages. **a** End-point dilution analyses were carried out on brain tissue homogenates from the cortex of sAD (Braak stage V or VI) and non-demented but with Braak stage II or III pathology samples. Results are shown as log SD_50_/mg brain tissue. **b** Lag time, determined by the assay time required to exceed ThT fluorescence values of the average + 100 standard deviations of the baseline fluorescence. Each symbol represents an individual well. Cross hatches and bars indicate the mean ± SD of the values at each dilution. Any data points beyond the assay endpoint (indicated by a red line) had positive ThT fluorescence values at or greater than 30 h. These data points were assigned a value of 30 to calculate mean ± SD

We tested if the Braak staging of preclinical and AD samples influenced the amount of AD seeds we were able to detect with AD RT-QuIC. We obtained brain tissue samples from the cortex of two non-demented samples with Braak stage II neuropathology and two samples at Braak stage III as well as Braak stage V and VI sAD cases. Brain tissue homogenates were used for end-point dilution analyses to determine seeding doses/mg tissue equivalents. sAD samples from Braak stage V and VI brain tissue had ~ 100-fold higher seeding activity than samples from Braak stage III and 10,000-fold higher seeding activity than samples from Braak stage II samples (Fig. 6). This indicated that increases in AD seeding activity might correlate with tau pathology burden in the tissue from which the homogenate is obtained or with a more advanced Braak stage.

### Detection of seeds in different brain regions, including those without immunohistochemically detectable tau deposits

**Fig. 7:**
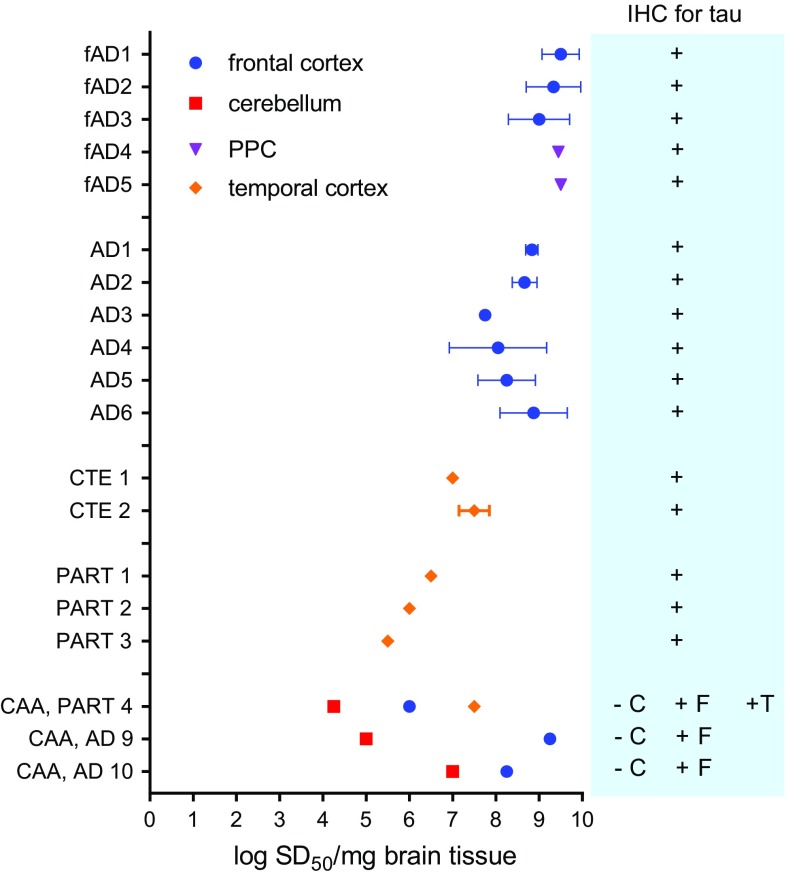
Seeding activity in brain regions with and without immunohistochemically visible 3R/4R tau deposits. Seeding activity measured in brain tissue homogenates derived from the frontal cortex, precuneus/posterior cingulate (PPC) cortex, temporal cortex and cerebellum from AD, CTE, and PART cases is indicated. Results are shown as log SD_50_/mg brain tissue (mean ± SD; *n* = 1–5 independent end-point dilution analyses for each case) and are originally plotted in Fig. 3 for all cases except select brain regions from PART 4 and AD 9 and 10 cases. One PART and two AD cases had neuropathological diagnoses of cerebral amyloid angiopathy (CAA) in the cerebellum and frontal cortex. Seeding activity was measured in homogenates from both the cerebellum, which lacks immunochemically visible tau deposits, and the frontal cortex. For the PART case, seeding activity was also determined for a homogenate derived from the temporal cortex. *AD* Alzheimer disease, *CTE* chronic traumatic encephalopathy, *PART* primary age-related tauopathy, *PPC* precuneus/posterior cingulate cortex, *C* cerebellum, *F* frontal cortex, *T* temporal cortex, *IHC* immunohistochemistry, *SD*_*50*_ seeding dose

We tested homogenates from frontal and cerebellar cortex from two AD cases and one PART case with evident cerebral amyloid angiopathy (CAA) (Fig. 7). As expected in the AD samples, the cerebellum had Aβ deposits in the blood vessel walls and no tau pathology, whereas the frontal cortex had both Aβ and tau pathology. The case of PART 4 had secondary diagnoses of CAA affecting the frontal and cerebellar cortex. As expected in this sample, the frontal and cerebellar cortex had Aβ deposits in the blood vessel walls and no tau pathology. Seeding activity was detected in the cerebellum of all three cases, albeit at levels 10–10,000-fold lower than the levels detected in the frontal cortex from the two AD cases. This suggests that AD RT-QuIC detected seeding activity even in brain regions without immunohistochemically detectable tau deposits. Data points from other AD samples, originally shown in Fig. 3 were plotted with the brain region analyzed as indicated. Seeding doses were comparable in AD brain homogenates derived from precuneus/posterior cingulate cortex (PPC), or frontal cortex regions, regions that are all known to have tau deposition at end-stage AD [3].

## Discussion

Here, we present a highly sensitive and selective assay for tau aggregates of AD and CTE. Recurring failures in therapeutic trials for AD have been attributed in part to insufficient abilities to identify the underlying cause(s) of disease and to quantify and differentiate relevant pathological oligomers or aggregates of tau and Aβ as biomarkers [22]. With respect to tau pathology alone, many different self-propagating “strains” of tau aggregates have been isolated and faithfully replicated in cell cultures which, when inoculated into transgenic mouse models, can cause distinct neuropathological lesions [7, 23, 31]. Consistent with this model of prion-like pathological tau strains, we previously demonstrated profound selectivity of the tau seeding activity of aggregates associated with PiD [37]. Here, we show that the tau seeding activities of AD and CTE are strikingly different from that of most cases of PiD and other diseases with different types of tau pathology, including those with predominant 4R tau aggregation. These differences were revealed by the choice of recombinant tau substrate, polyanionic cofactors, and other reaction conditions, but presumably reflects the underlying conformation(s) of the tau aggregates that accumulate preferentially in the context of AD. Indeed, the recent cryo-EM-based structures of AD and PiD tau filaments have indicated marked differences in their amyloid cores [12–14]. As has been documented with distinct TSE prion strains, we provide evidence that the distinct structures of tau aggregates that accumulate in different diseases have self-propagating activities that were readily distinguished by their relative abilities to seed the polymerization of various tau substrates under suitable conditions.

Although AD RT-QuIC usually detected orders of magnitude higher seeding activities in AD and CTE brain samples compared to those of various human non-AD control samples, it also detected weaker activities in the latter that were well above those in tau-free KO mouse brains (Figs. 1, 2, 3). One possible explanation for this is that although the predominant tau aggregates in the non-AD brains with tau pathology are qualitatively distinct from those in AD, they can be weakly active as seeds under the AD RT-QuIC conditions. Alternatively, non-AD brains might contain mixed types of tau aggregates, with only a small minority being AD-like (3R/4R) and capable of seeding AD RT-QuIC reactions. Our analyses suggest that seeding activity may be influenced by the load of tau pathology in the regions analyzed and that, as a consequence, it might also increase in cases with higher Braak stage. Seeding activity detected in cases with another 3R/4R pathology, PART, was largely comparable to that measured in many of the non-AD brains. Because PART is most frequently observed in elderly individuals, the low levels of seeding activity detected by the AD RT-QuIC across the samples analyzed for this study may reflect the detection of different levels of tau accumulation as a result of age as occurs in PART. Still, another possibility is that there is a yet unidentified non-tau component(s) of human, but not mouse, brain that can accelerate unseeded (spontaneous) nucleation and fibrillization of the recombinant tau substrate molecules. However, this latter explanation seems unlikely because, in the case of DLBD, at least, the seeding activity was, like AD tau filaments, sarkosyl-insoluble and protease resistant. The fact that the control brains were negative for abnormal tau deposits by immunohistochemical analysis does not establish that they lacked any abnormal tau that could be detectable by AD RT-QuIC, because the latter assay is likely to be more sensitive, and/or less affected by localized sampling artifacts, than immunohistochemistry. Regardless, as the seeding activity in AD brain tissues is many fold higher than in most non-AD specimens, comparisons of AD RT-QuIC lag phases and/or end-point dilutions should allow identification of AD brain tissue with high, but perhaps not absolute, specificity. Notably, the AD RT-QuIC detected seeding activity in two CTE cases at levels comparable to weaker examples of the sAD cases (i.e., ~ 10^−7^ dilutions). This suggests that AD RT-QuIC is capable of detecting 3R/4R tau seeds, not necessarily only those derived from AD.

From a practical perspective, the ability to selectively detect and quantitate AD and CTE tau aggregates by AD RT-QuIC may be useful in both research and diagnostics. As a research tool, the ultrasensitive nature of the AD RT-QuIC makes it particularly useful for the identification of when and where such aggregates accumulate. For example, use of AD RT-QuIC could be used to carefully elucidate which brain regions, at different stages of disease, contain tau seeds to aid interpretation of experimental attempts to manipulate or halt the spread of pathological tau aggregation throughout the brain. Importantly, further development of AD RT-QuIC for use with diagnostically relevant specimens, such as cerebrospinal fluid, could provide an etiological tau biomarker to help definitive diagnosis and selection of patient cohorts for clinical trials, in addition to longitudinal evaluation of AD tau levels in response to treatments. As noted above, a seed amplification assay, the Aβ PMCA, has already been reported for the Aβ oligomers of AD [38]. Combining the results of these and perhaps other protein seed amplification assays with other clinical and neuropathological indices should help to refine investigators’ abilities to use tau and Aβ seeds as biomarkers in elucidating the underlying causes of AD and related protein misfolding diseases, and better assess effects of potential therapeutics. Further studies will be required to assess the clinical significance, if any, of the lower levels of AD RT-QuIC tau-seeding activity that we have detected in many of the non-AD cases.

## Electronic supplementary material

Below is the link to the electronic supplementary material.

Supplementary material 1 (PDF 2177 kb)
