# Supplementary material for: Seeding selectivity and ultrasensitive detection of tau aggregate conformers of Alzheimer disease
Source: Acta Neuropathol. 2018 Dec 20;137(4):585–98. doi: 10.1007/s00401-018-1947-3 (PMC6426988; doi:10.1007/s00401-018-1947-3)
Supplement: Supplementary file 1 — Supplementary material 1 (PDF 2177 kb) [file 401_2018_1947_MOESM1_ESM.pdf]

Supplementary material for

Seeding selectivity and ultrasensitive detection of tau aggregate conformers of Alzheimer disease

Acta Neuropathologica

Allison Kraus[1*], Eri Saijo[1*], Michael A. Metrick II.[1*], Kathy Newell[2], Christina J. Sigurdson[3], Gianluigi Zanusso[4], Bernardino Ghetti[5] and Byron Caughey[1#]

[1]LPVD, Rocky Mountain Laboratories, NIAID, NIH, Hamilton, MT 59840 USA.

[2] Department of Pathology and Laboratory Medicine, University of Kansas School of Medicine, Kansas City, KS, USA

[3] Department of Pathology, UC San Diego, La Jolla, CA, USA

[4] University of Verona, Verona, Italy.

[5] Indiana University School of Medicine, Indianapolis, IN, USA

*These authors contributed equally.

[#]Corresponding author: bcaughey@nih.gov; TEL: +1 406 363 9264; FAX: +1 406 363 9286

Online Resource Table 1.

Tauopathy classific.	1° (2°, 3°, 4°) Diagnosis	Brain region	Sex	AOD	PMI (Hrs)	Brain Wt.(g)	Braak score	tau RT-QuIC seed concentration (log SD_{50}/mg tissue) ± SE					Average ± SD	inter-assay CV
								Expt 1	Expt 2	Expt 3	Expt 4	Expt 5		
3R/4R	sAD 1[a]	F	M	58	13	NA	VI	9.00±0.29	8.75±0.38	8.75±0.38	-	-	8.83±0.14	0.016
	sAD 2[a]	F	F	80	26	1007	VI	9.00±0.41	8.50±0.46	8.50±0.35	-	-	8.67±0.29	0.033
	sAD 3[a]	F	M	70	9	1390	VI	7.75±0.25	7.75±0.46	7.75±0.25	-	-	7.75±0	0.000
	sAD 4[a]	F	F	72	24	U	VI	7.50±0	7.25±0.25	10.00±0.36	7.50±0	8.0±0.29	8.05±1.12	0.140
	sAD 5[a]	F	M	80	30	U	VI	8.75±0.38	8.50±0.29	7.50±0	-	-	8.25±0.66	0.080
	sAD 6[a]	F	F	76	24	U	VI	9.75±0.46	9.25±0.46	8.00±0.25	8.5±0.38	-	8.88±0.78	0.088
	sAD 7 (CM)[b]	Cx	M	80	3	1000	VI	8.50±0	-	-	-	-	8.50	
	sAD 8 (CM)[b]	Cx	F	78	2	750	VI	8.50±0.35	-	-	-	-	8.50	
	sAD 9 (CAA) (regional neuro-path, same brain as CAA 9)[c]	F	F	73	16.3	1110	VI	9.25±0.25	9.25±0.25	-	-	-	9.25±0	0.000
	sAD 10 (CAA) (regional neuro-path, same brain as CAA 10)[c]	F	M	85	9.6	1300	V	8.25±0.38	-	-	-	-	8.25	
	sAD 11[a]	F	F	82	19	1049		7.50±0.36	8.00±0.29	8.25±0.43	-	-	7.92±0.38	0.048
	fAD-A431E 1[a]	F	M	44	U	U		9.25±0.54	9.25±0.38	10.00±0.38	-	-	9.5±0.43	0.046
	fAD-A431E 2[a]	F	F	43	4	U		10.00±0.36	8.75±0.25	9.25±0.38	-	-	9.33±0.63	0.067
	fAD-A431E 3[a]	F	U	U	U	U		9.50±0.48	8.50±0	-	-	-	9±0.71	0.079
	fAD-L435F 4[d]	PPC	F	56	12	926	VI	9.45±0.25	-	-	-	-	9.45	
	fAD-A431E 5[d]	PPC	F	55	8	720	VI	9.50±0	-	-	-	-	9.50	
	CTE 1 (DLB)[c]	T	M	78		1480		7.00±0.5	7.00±0.46	-	-	-	7.00±0	0.000
	CTE 2[c]	T	M	67		1240		7.25±0.25	7.75±0.38	-	-	-	7.50±0.35	0.047
	PART 1 (LBD, CVD)[a]	T	F	77	3.5	1063	N/A	6.50±0.35	-	-	-	-	6.50	
	PART 2 (ALS, CVD)[a]	T	M	62	3.5	1447	N/A	6.00±0.35	-	-	-	-	6.00	
	PART 3 (small vessel disease, TDP43, CVD)[a]	T	M	65	4.5	1103	N/A	5.50±0.35	5.50±0.41	-	-	-	5.50±0	0.00
	PART 4 (regional neuropath, same brain as CAA 4)[c]	T	F	88	7.9	1140		7.50±0	-	-	-	-	7.50	
	CAA 4 (regional neuropath, same brain as PART 4)[c]	F	F	88	7.9	1140		6.00±0.29	-	-	-	-	6.00	
	ND 05-12 (CAA)[b]	Cx	F	88	2	1160	III	5.75±0.56	-	-	-	-	5.75	
	ND 06-15[b]	Cx	F	87	3	1260	III	6.50±0.54	-	-	-	-	6.50	
	ND 03-41 (CWMR)[b]	Cx	M	89	3	1440	II	4.25±0	-	-	-	-	4.25	
	ND 06-21[b]	Cx	F	73	3	1240	II	5.00±0.29	-	-	-	-	5.00	
4R	PSP 3 (CVD)[a]	F	M	65	8	1175		5.25±0.38	5.50±0.38	-	-	-	5.38±0.18	0.033
	PSP 4 (SC)[a]	F	M	67	4	1480		6.25±0.25	5.75±0.25	-	-	-	6±0.35	0.059
	PSP 5 (CVD)[a]	F	M	76	6	1381		4.25±0.25	4.25±0.25	-	-	-	4.25±0	0.000
	CBD 1[a]	F	F	51	10	U		6.00±0.29	5.50±0.36	-	-	-	5.75±0.35	0.061
	CBD 3 (CVD)[a]	F	M	65	17	1200		6.50±0	6.75±0.36	-	-	-	6.63±0.18	0.027
	AGD 1 (CVD)[a]	F	M	91	2	1175		5.75±0.25	6.25±0.38	-	-	-	6±0.35	0.059
	AGD 2 (HS)[a]	F	M	86	110	1330		5.50±0.25					5.5	
	FTDP-17 (MAPT) 3[a]	F	F	54	11	1122		6.00±0.29	4.75±0.29	6.25±0.25	-	-	5.67±0.8	0.142

3R	PiD 1 (CVD)[a]	F	M	56	9	1032		6.25±0.25	5.50±0.29	-	-	-	5.88±0.53	0.090
	PiD 2 (β-amyloid pathology, CVD)[a]	F	M	64	3	1003		8.00±0.46	6.25±0.25	5.75±0.25	-	-	6.67±1.12	0.177
	PiD 4[a]	F	M	70	U	U		5.00±0.29	-	-	-	-	5	
IHC no tau	PD 1[e]	F	M	73	U	U		5.25±0.25	5.50±0.29	-	-	-	5.38±0.18	0.033
	PD 2[e]	F	M	61	U	U		4.25±0.25	5.50±0.29	5.50±0.36	-	-	5.08±0.72	0.142
	SC 1[a]	F	F	76	4	1240		5.00±0.29	5.50±0.36	-	-	-	5.25±0.35	0.067
	SC 3[a]	F	F	81	25	1100		5.25±0.38	6.00±0.29	4.75±0.25	-	-	5.33±0.63	0.118
	CVD 1[a]	F	F	53	19	1192		3.75±0.25	4.50±0.35	4.50±0	-	-	4.25±0.43	0.102
	CVD 3 (SC)[a]	F	F	77	6	1112		3.50±0	4.00±0.29	4.50±0.29	2.75±0.25	-	3.69±0.75	0.202
	CVD 4 (SAH, SC)[a]	F	F	86	U	1050		5.50±0.41	2.50±0	5.25±0.38	-	-	4.42±1.66	0.377
	DLBD 1 (SC, CVD)[a]	F	M	80	12	1285		6.50±0	6.50±0.36	7.00±0.35	-	-	6.67±0.29	0.043
	DLBD 4 (CVD, SC)[a]	F	M	71	8	1395		5.25±0.36	5.25±0.25	-	-	-	5.25	0.000
	FTLD-TDP 1 (CVD)[a]	F	F	50	6	754		5.25±0.25	-	-	-	-	5.25	
	FTLD-TDP-Type A 4 (CVD)[a]	F	F	65	5	886		3.25±0.25	4.75±0.38	-	-	-	4±1.06	0.265
	ALS 1 (SC, CVD)[a]	F	M	72	2	1376		5.00±0.36	4.75±0.38	-	-	-	4.875±0.18	0.36
	CAA 9 (regional neuropath, same brain as AD 9)[c]	C	F	73	16.3	1110		5.00±0.29	-	-	-	-	5.00	
	CAA 10 (regional neuropath, same brain as AD 10)[c]	C	M	85	9.6	1300		7.00±0.46	-	-	-	-	7.00	
	CAA 4 (regional neuropath, same brain as PART 4)[c]	C	F	88	7.9	1140		4.25±0.38	-	-	-	-	4.25	
	tau KO	Whole	U	U	U	U								

Neuropathological diagnoses were provided for the indicated cases by [a]Dr. Bernardino Ghetti, [b]Dr. Thomas G. Beach, [c]Dr. Kathy Newell, [d]Dr. Lawrence A. Hansen, [e]Dr. Roscoe Atkinson

Abbreviations:

AGD, Argyrophilic grain disease
AD, Alzheimer disease
ALS, Amyotrophic lateral sclerosis
CAA, cerebral amyloid angiopathy
CM, cerebral malformation
CVD, cerebrovascular disease
CWMR, cerebral white matter rarefaction
DLBD, diffuse Lewy body disease
FTDP-17, Frontotemporal dementia and parkinsonism linked to chromosome 17
FTLD-TDP, Frontotemporal lobar degeneration with TDP-43
HS, Hippocampal sclerosis
PD, Parkinson disease
PiD, Pick diseae
PART, primary age-related tauopathy
PSP, progressive supranuclear palsy
SAH, sub-arachnoid hemorrhage
SC, senile change

AOD, age of death
N/A, non-applicable
tau KO, human and mouse tau knockout mouse
U, unavailable
PMI, post-mortem interval

Brain region:

F, frontal cortex
T, temporal cortex
C, cerebellum
Cx, cortex
H, hippocampus
PPC, precuneus/posterior cingulate cortex

Online Resource Fig 1.

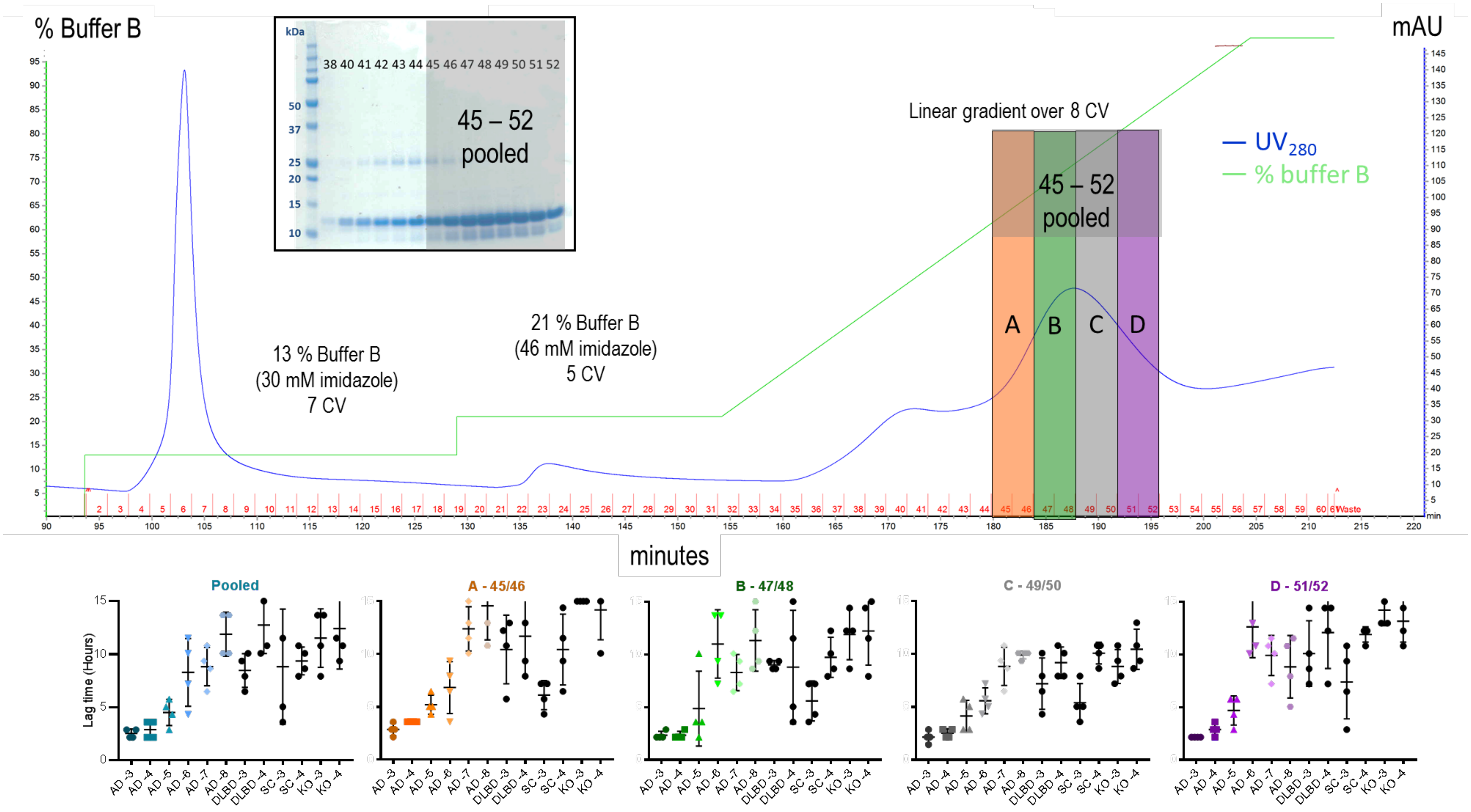

Online Resource Fig 1. Purification of AD RT-QuIC assay ready τ306 substrate. Contaminants of the AD RT-QuIC are eluted from the FF His column with two steps of 13% and 21% buffer B prior to elution of τ306 over a linear gradient of 21% to 100% buffer B over 8 column volumes. Fractions of τ306 were tested in a 384 well plate for the reproducibility and sensitivity of AD detection. Based on this and the purity of the substrate assessed by Coomassie blue gel analysis (inset), fractions 45-52 were pooled, precipitated with acetone before guanidine monomerization and size-exclusion desalting to yield pure monomeric τ306.

Online Resource Fig 2

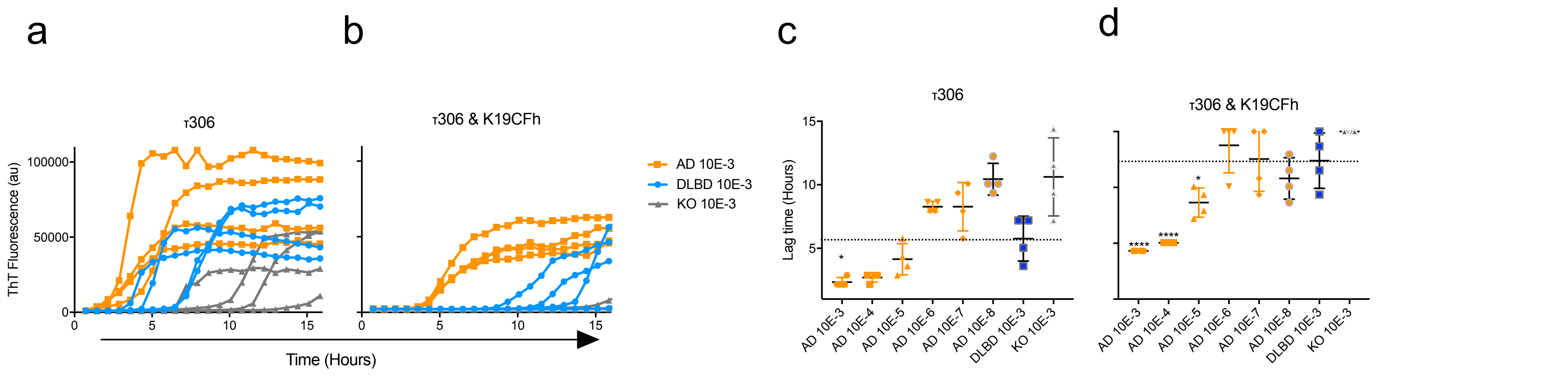

Online Resource Fig 2. Comparison of τ306 alone or mixed with K19CFh as substrates for detection of AD-associated tau seeding activity.

a. τ306 alone was tested as a substrate for RT-QuIC reactions seeded with AD or DLBD brain homogenates in a 96 well plate. Mouse tau KO brain homogenate was used a tau-free control. AD brain homogenates modestly accelerated amyloid formation. **b.** Inclusion of K19CFh in a 3:1 stoichiometric ratio with τ306 gave improved discrimination of AD brain homogenate dilutions from DLBD and tau KO brain homogenates. **c.** Lag time analyses showed use of τ306 substrate alone only allowed significant discrimination of 10^{-3} dilutions of AD brain homogenate from DLBD brain homogenate, which lacked histologically detectable tau pathology. **d.** Inclusion of K19CFh with τ306 (3:1 stoichiometry) allowed significant discrimination of 10^5 SD_{50}/mg with AD brain homogenate prior to positive ThT fluorescence readings in the presence of DLBD brain homogenate. One way ANOVA, $F(7,24)=3.36$, *$p<0.01$; $F(7,24)=19.49$, ****$p<0.0001$. For comparative purposes, the assay endpoint is shown as 15 h, and data points shown at 15 h had positive ThT fluorescence values at or greater than 15 h. These data points were assigned a value of 15 for statistical calculations, including mean ± S.D.

Online Resource Fig 3.

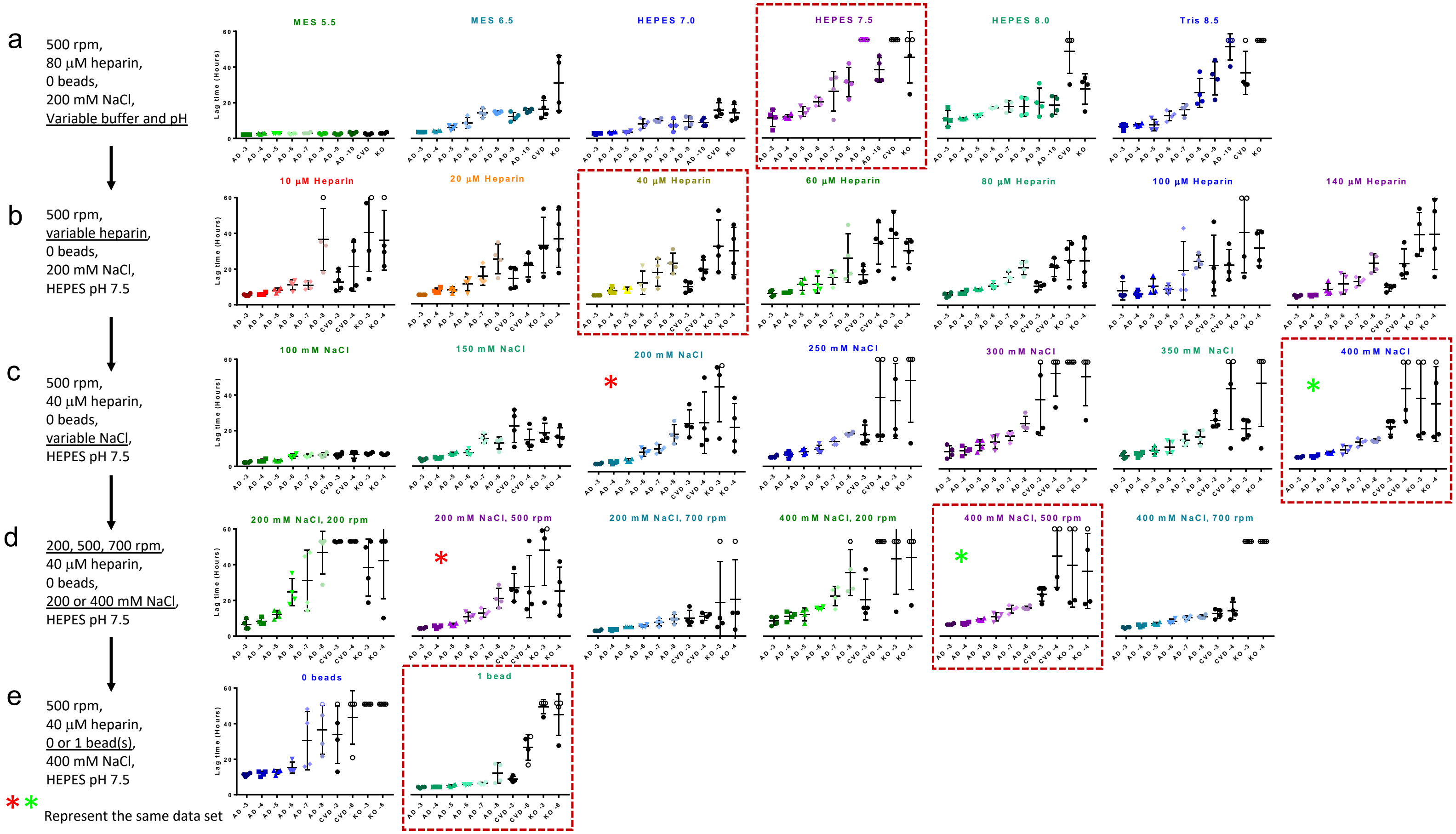

Online Resource Fig 3. Optimization of AD RT-QuIC assay conditions. Conditions were optimized using a 1:3 stoichiometric ratio of τ306:K19CFh. Iterative optimizations were carried out in the following order: a) pH, b) heparin concentration, c) salt concentration, d) shaking speed during incubation at 200 mM and 400 mM NaCl, as well as e) the inclusion of one silica bead in the assay. For each variable tested, the final condition selected is indicated with a box. Green and red asterisks indicate the same data sets, shown twice for comparison. Assay endpoints ranged from 51 – 60 hours. Wells that had not yet exceeded threshold fluorescence at the end of the assay are shown as open symbols and included in mean ± SD calculations for comparative purposes. Conditions were chosen to optimize selectivity for AD against CVD-seeded wells while delaying spontaneous fibril formation in KO-seeded wells.

Online Resource Figure 4.

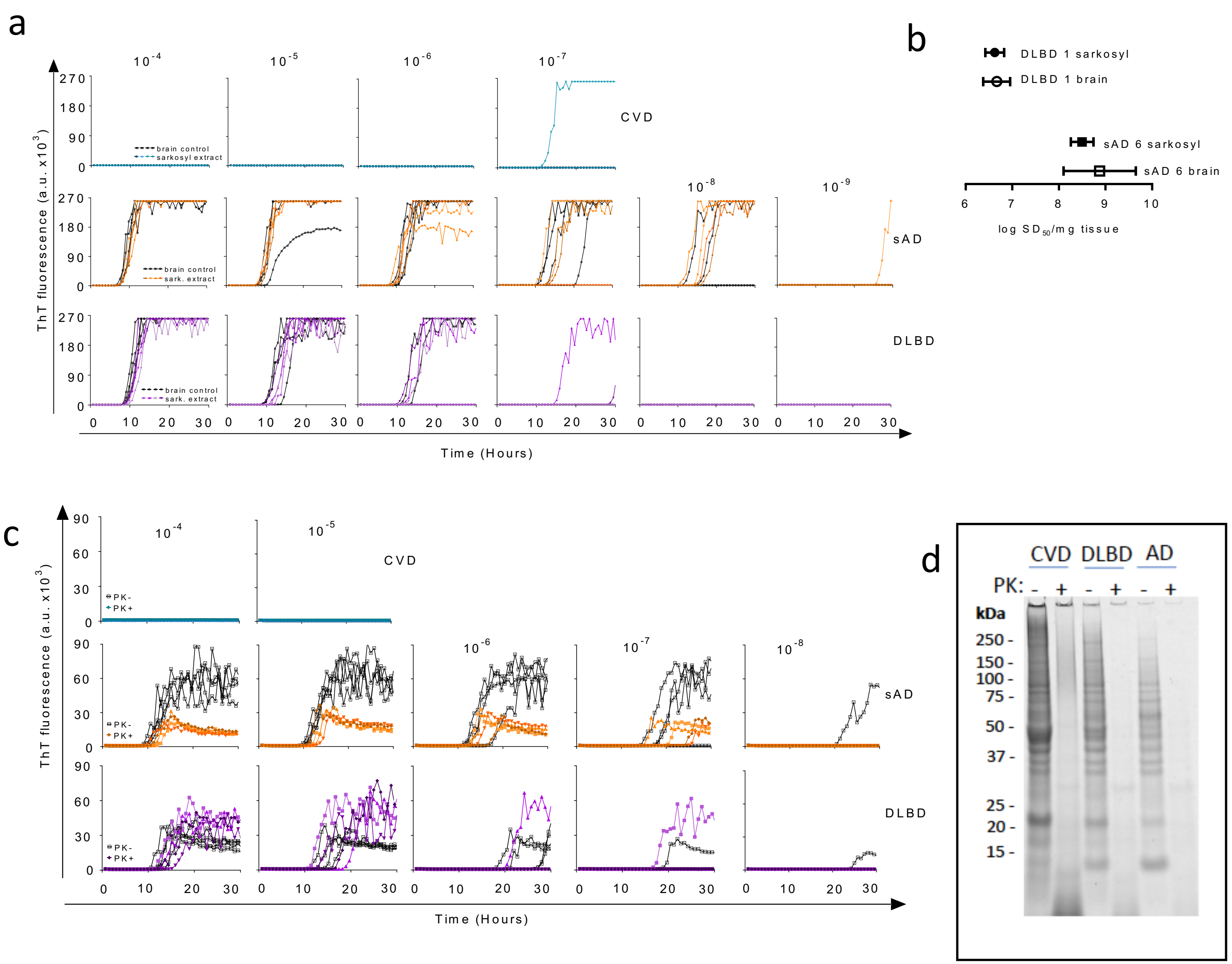

e

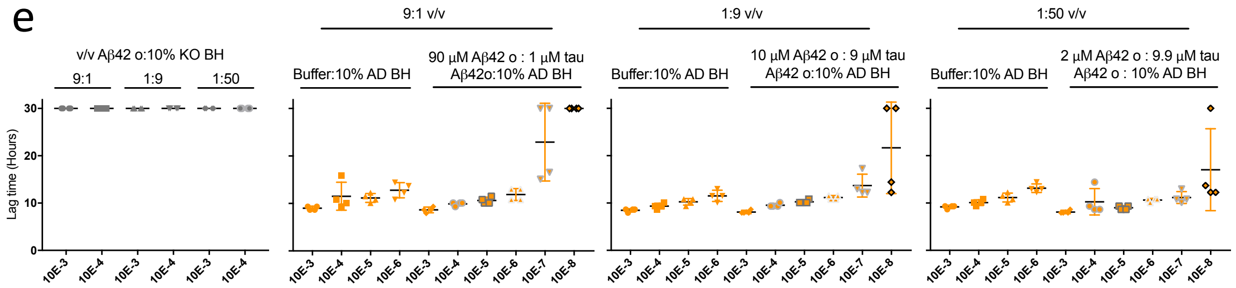

Online Resource Fig 4. Seeding activity is largely derived from sarkosyl-insoluble, protease resistant tau and is not significantly influenced by Aβ42 oligomers.

a. Brain equivalents of Sarkosyl-insoluble tau were analyzed in parallel to brain homogenates from the matching brain in the AD RT-QuIC. Each curve represents ThT fluorescence from an individual well, run in quadruplicate. **b.** The mean log SD_{50}/mg ± S.E. is shown for DLBD and AD brain homogenates (brain) and sarkosyl insoluble extracts (sarkosyl). **c**. CVD non-tauopathy and DLBD and AD brain homogenates were treated with proteinase K prior to analysis by RT-QuIC and compared to non-PK treated brain homogenates by endpoint-dilution. Each curve represents ThT fluorescence from an individual well, run in quadruplicate. **d.** Proteinase-K (PK+) treated and non-treated (PK-) CVD, DLBD, and AD brain homogenates used for RT-QuIC analyses were run on an SDS-PAGE gel and analyzed by Deep purple total protein stain to confirm PK digestion. **e**. Aβ42 oligomers (Aβ42 o) were mixed at different molar ratios as indicated with mouse tau KO or AD brain homogenate samples and these mixtures were used for dilution analyses in the AD RT-QuIC reactions. Control reactions containing mouse tau KO brain homogenates were matched v/v to the AD brain homogenate samples. Molarity of tau was calculated based on the assumption of ~10 μM total tau in an AD brain. Each point indicates the lag time of an individual well with mean ± S.D. indicated by the cross hatches and bars. Data points beyond the assay endpoint at 30 h were assigned a value of 30 for comparative purposes. Addition of oligomers did not significantly influence the lag phases of AD brain-seeded fluorescence readings. BH, brain homogenate.

Online Resource Fig 5.

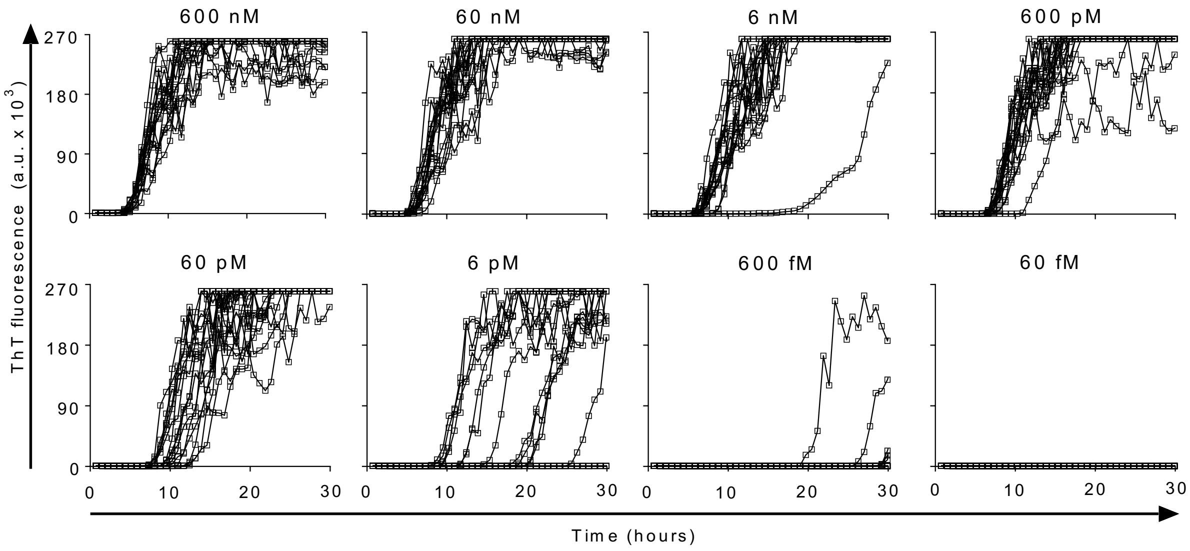

Online Resource Fig 5. Endpoint dilution of synthetic AD brain-seeded τ306 and K19CFh fibrils in AD RT-QuIC reactions. Synthetic recombinant τ306 & K19CFh fibrils were collected after 60 h of continuous shaking at 1000 rpm, 37 °C and quantitated using gel analysis against a standard curve of τ306 & K19CFh recombinant protein. Endpoint dilution analysis in the AD RT-QuIC was used to determine the molar analytical sensitivity of the synthetic fibrils. Each panel represents the ThT fluorescence readings of sixteen replicate wells at the designated fibril concentration, analyzed over four individual experiments.
